# Atypical Postoperative Presentation of Malignant Hyperthermia in an Elderly Patient Following Transcatheter Aortic Valve Replacement

**DOI:** 10.7759/cureus.94285

**Published:** 2025-10-10

**Authors:** Matthew W Knick, Benton Nanners, Mir Ali Abbas Khan, Arpan Kohli

**Affiliations:** 1 Anesthesiology, West Virginia University School of Medicine, Morgantown, USA

**Keywords:** dantrolene, hypermetabolic crisis, malignant hyperthermia, postoperative complications, transcatheter aortic valve replacement (tavr)

## Abstract

We report an unusual case of an 82-year-old female who developed acute respiratory distress, tachycardia, diaphoresis, and hypertension in the post-anesthesia care unit (PACU) following transcatheter aortic valve replacement (TAVR), which improved significantly after initiation of dantrolene. Given the patient's atypical age, delayed symptom onset, and notable improvement following dantrolene administration, we present an atypical case in which malignant hyperthermia (MH) could not be ruled out based on clinical progression. This case underscores the importance of maintaining a high index of suspicion for MH in atypical presentations and highlights the critical role of timely diagnosis and intervention.

## Introduction

Malignant hyperthermia (MH) is a rare and potentially life-threatening hypermetabolic disorder triggered by certain anesthetic agents, including succinylcholine and volatile anesthetics. The incidence of MH is estimated at approximately one in 100,000 anesthetic administrations, with a higher prevalence in males and pediatric patients, who account for 42%-52% of cases [1,2]. MH is associated with mutations in the ryanodine receptor 1 (RYR1) or dihydropyridine (DHP) receptors in skeletal muscle, leading to excessive calcium release into the sarcoplasm upon exposure to triggering agents. This dysregulation results in sustained muscle contractions, manifesting as hypercarbia, tachycardia, muscle rigidity, and hyperthermia. As the disorder progresses, patients may develop mixed respiratory and metabolic acidosis due to anaerobic metabolism, accompanied by hyperkalemia and myoglobinuria secondary to rhabdomyolysis [2,3]. Development of MH typically occurs within 0-40 minutes [4]. The MH clinical grading scale is one tool used for the assessment of MH [5].

Dantrolene is the primary treatment for MH, acting by binding to the RYR1 receptor and thereby inhibiting further calcium release from the sarcoplasmic reticulum. The introduction of dantrolene, along with advancements in patient monitoring, has significantly reduced the mortality rate of MH from nearly 80% to approximately 10% [2,3]. Although most MH cases occur intraoperatively, about 1.9% manifest in the postoperative period [4]. Postoperative MH presents unique challenges due to its rarity, decreased patient monitoring, and a broad differential diagnosis. Postoperative MH must be differentiated from other acute postoperative conditions, such as febrile non-hemolytic transfusion reactions, sepsis, thyroid storm, serotonin syndrome, and neuroleptic malignant syndrome (NMS). Of these, NMS and serotonin syndrome are related conditions with a similar presentation as MH and are differentiated from MH due to exposure to antipsychotic and serotonergic medication, respectively. Elderly patients have an increased risk of these conditions due to comorbid conditions, polypharmacy, and cardiac disease, making the diagnosis of MH challenging.

This case report presents a medically challenging scenario involving an 82-year-old female who exhibited symptoms consistent with MH approximately 50 minutes after cessation of sevoflurane during the postoperative period. The patient had no documented history of exposure to known MH-triggering agents or personal/familial anesthetic complications. Despite the absence of definitive diagnostic criteria, the significant clinical improvement following administration of dantrolene suggests that MH could not be ruled out.

## Case presentation

An 82-year-old female patient, weighing 74 kg and measuring 1.56 m in height, was scheduled for transcatheter aortic valve replacement (TAVR) due to worsening fatigue and dyspnea from severe aortic stenosis over the past year. Her medical history included atrial fibrillation, heart failure with preserved ejection fraction (50%), pulmonary hypertension, hypertension, bilateral carotid artery stenosis, cerebrovascular accident, Barrett’s esophagus, and a hiatal hernia. Her surgical history included cardiac catheterization, colonoscopy, hysteroscopy with dilation and curettage, esophagogastroduodenoscopy, and right carotid endarterectomy. She had no personal or familial history of anesthesia-related complications. Preoperatively, her medications included warfarin (held three days before surgery), furosemide, magnesium oxide (both held on the morning of surgery), pantoprazole, and digoxin. Laboratory results were unremarkable, except for an elevated BNP of 122 pg/mL. The most recent electrocardiogram showed atrial fibrillation, and an echocardiogram revealed moderate-severe mitral stenosis, moderate aortic stenosis with mild regurgitation, severe pulmonary hypertension, and a left ventricular ejection fraction (LVEF) of 55-60%. On the day of surgery, her vital signs were blood pressure of 159/80 mmHg, heart rate of 68 beats per minute, respiratory rate of 14 breaths per minute, and oxygen saturation of 99%. Physical examination noted a Mallampati class II airway, full neck range of motion, fair mouth opening, peripheral edema, and decreased breath sounds.

**Figure 1:**
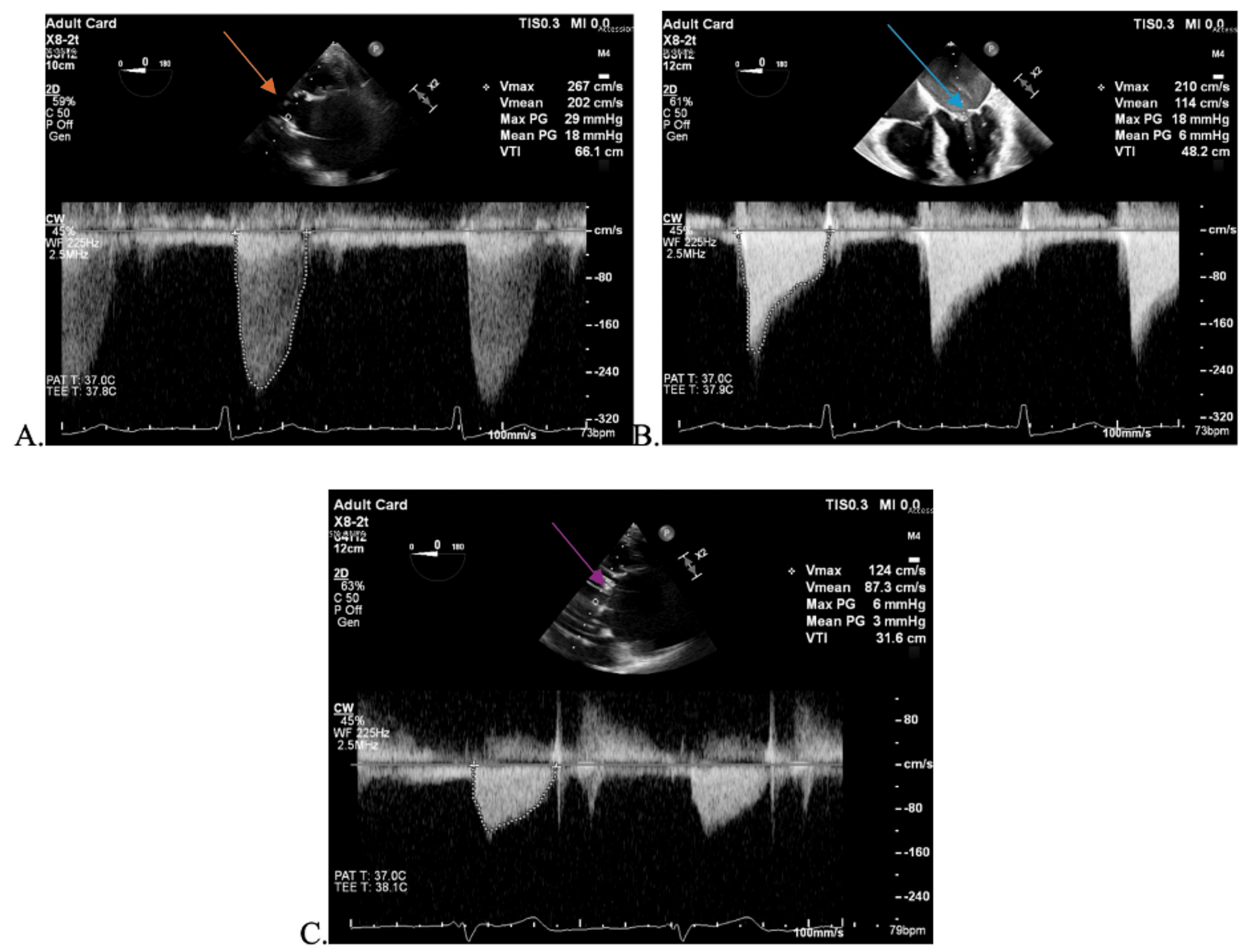
Focused TEE exam performed in the setting of transcatheter aortic valve replacement (TAVR). Preprocedural echo showed normal biventricular size with left ventricular ejection fraction (LVEF) estimated at 35% with normal RV function. (A) Mild AI with a mean aortic valve gradient of 18 mmHg. The red arrow indicates the aortic valve. (B) Mild MR with moderate MS with a mean gradient of 6 mmHg. The blue arrow indicates the Mitral valve. (C) Post procedure, aortic valve was replaced with a stented bioprosthetic, indicated by a purple arrow, mean gradient of 3 mmHg, mild paravalvular leak, no pericardial or pleural effusion no aortic dissection. This highlights the patient's procedure before and after, showing successful placement of a bioprosthetic.

Anesthetic induction was achieved with 100 mcg fentanyl, a 100 mg dose of 2% lidocaine, 150 mg propofol, and 100 mg rocuronium, followed by endotracheal intubation using a size 3 Macintosh blade and a 7.0 endotracheal tube. Monitoring included an arterial line, pulse oximetry, electrocardiography, and non-invasive blood pressure measurement. Immediately post-induction, her vital signs were blood pressure of 150/102 mmHg, heart rate of 67 beats per minute, oxygen saturation of 100%, end-tidal CO_2_ (EtCO2) of 27 mmHg, and temperature of 35.5°C. General endotracheal anesthesia was selected to facilitate placement of a transesophageal echocardiogram (TEE) probe, as requested by the surgical team (Figure 1).

The TEE probe was inserted following intubation. Anesthesia was maintained with 2.0% sevoflurane supplemented by propofol boluses for a total anesthetic duration of 78 minutes. Throughout the procedure, the patient remained normothermic and hemodynamically stable, with EtCO_2_ values consistently in the low 30s. Prior to extubation, her vital signs were blood pressure of 154/72 mmHg, heart rate of 76 beats per minute, EtCO_2_ of 38 mmHg, and temperature of 35.5°C. Once extubation criteria were met, the endotracheal tube was removed, and the patient was transported to the post-anesthesia care unit (PACU) on a simple facemask, where her initial vital signs were within normal limits.

**Figure 2:**
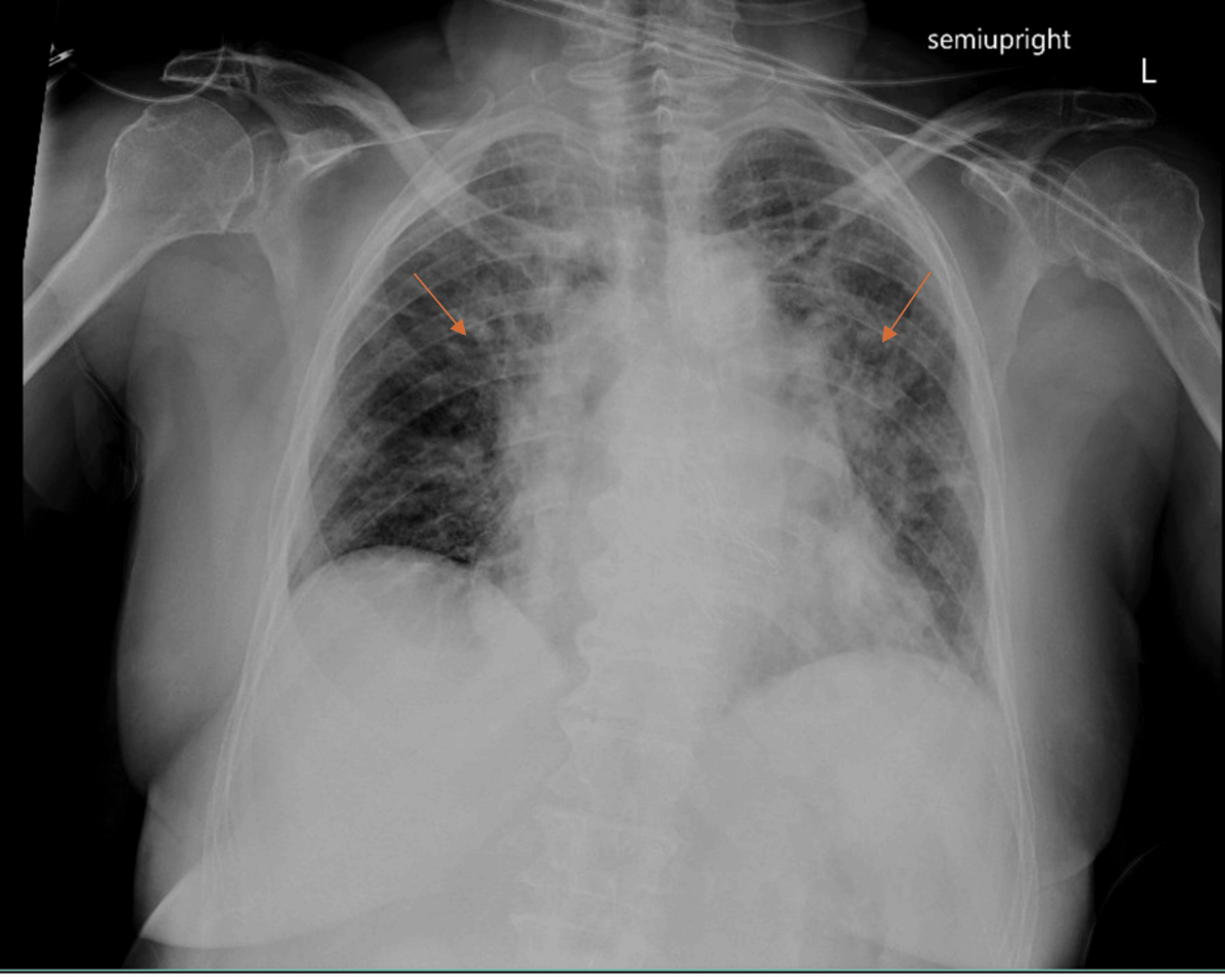
Chest X-ray following transcatheter aortic valve replacement (TAVR). Postsurgical changes from TAVR. Probable mild pulmonary edema/fluid overload (red arrows), accentuated by lung hypoinflation. This shows no acute finding to suggest this patient's constellation of symptoms.

Approximately 50 minutes after cessation of sevoflurane, the anesthesia team was urgently paged to the PACU due to the patient's acute distress. She exhibited dyspnea and diaphoresis with blood pressures exceeding 200/100 mmHg and a heart rate over 100 beats per minute. She remained normothermic per rectum. Lung auscultation revealed clear breath sounds bilaterally, and chest X-ray showed no acute cardiopulmonary abnormalities (Figure 2).

**Figure 3:**
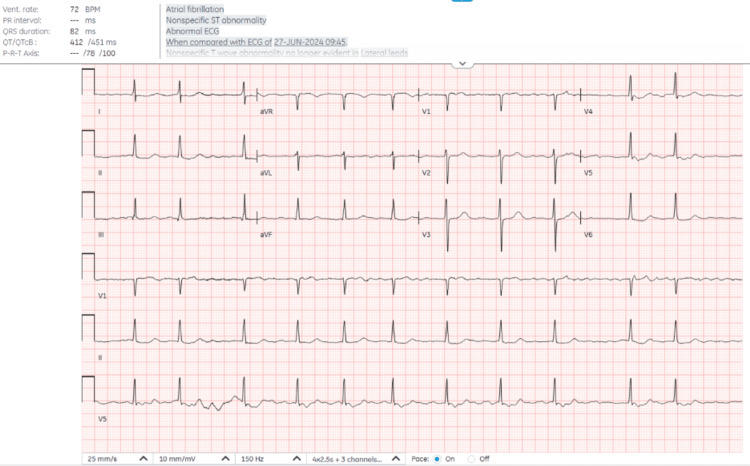
Post-operative ECG following transcatheter aortic valve replacement (TAVR). ECG atrial fibrillation with nonspecific ST abnormality when compared to pre-op ECG nonspecific T-wave abnormality no longer evident in lateral leads. QT has lengthened. There is no acute finding to suggest the clinical constellation in this patient.

ECG showed no significant deviation from the preoperative study (Figure 3).

Point-of-care ultrasound confirmed her baseline cardiac status. Arterial blood gas analysis revealed mixed respiratory and metabolic acidosis, with a pH of 7.14, pCO_2_ of 75 mmHg, pO_2_ of 70 mmHg, and bicarbonate of 20.5 mmol/L. Initial management with nitroglycerin and esmolol resulted in minimal blood pressure reduction. A clevidipine infusion was started at 2 mg/hour; however, the patient’s vitals continued to fluctuate. CK was 52. The patients' extremities did not exhibit any signs of clonus or lead-pipe rigidity on exam. A preliminary differential diagnosis included MH, hypertensive emergency, serotonin syndrome, and NMS.

Given the rapid onset of symptoms, refractory hypertension, and lack of medication history suggesting NMS or serotonin syndrome, the diagnosis of MH could not be ruled out. Additionally, her MH clinical grading score was 56. The decision was made to contact the MH hotline. Following consultation, dantrolene was administered, initially 90 mg and then an additional 60 mg. Within five minutes, there was a marked improvement in the patient’s condition, as evidenced by a pH of 7.24, pCO_2_ of 49 mmHg, pO_2_ of 78 mmHg, and bicarbonate of 19.7 mmol/L on repeat arterial blood gas. Her dyspnea, diaphoresis, hypertension, and tachycardia significantly improved, and her mentation returned to baseline. She was subsequently transferred to the cardiovascular intensive care unit (CVICU) for further monitoring and management.

In the CVICU, the patient developed transient slow atrial fibrillation, with a heart rate of 30 beats per minute, resulting in hypotension. The dantrolene infusion was discontinued as her pCO_2_ normalized. Her bradycardia resolved without further intervention. The patient was gradually weaned from bilevel positive airway pressure (BiPAP) to nasal cannula and eventually to room air prior to discharge on postoperative day three. Genetic testing or caffeine halothane testing was recommended for future anesthetic planning; however, the patient did not obtain these confirmatory tests.

## Discussion

MH is a rare and life-threatening hypermetabolic disorder triggered by exposure to specific anesthetic agents. This case highlights the diagnostic complexity that arises when clinical features do not perfectly align with the typical presentation of MH and symptoms overlap with other critical postoperative conditions.

Several factors contributed to the diagnostic challenge. The patient’s demographic profile was atypical. Data from the North American Malignant Hyperthermia Registry (1987-2006) indicate that young males account for approximately 74.86% of MH cases [6]. In contrast, our patient was an 82-year-old female, representing a significant deviation from the typical demographic profile associated with MH. Additionally, although more than half of MH patients lack a prior anesthesia-related history and nearly 90% have a negative family history [6], this patient’s prior surgical exposure without complications further reduced the likelihood of presenting with MH for the first time.

Symptom onset was also atypical for MH. While the majority of MH episodes occur intraoperatively, only about 2% present after anesthesia discontinuation, typically within 0-40 minutes [4]. In this case, symptoms appeared approximately 50 minutes after cessation of sevoflurane. One factor that strengthened the diagnosis of MH is the MH clinical grading scale [5]. The MH clinical grading scale is a valuable tool for assessing the likelihood of MH [5]. Our patient’s score of 56 on this scale indicated a high probability of MH.

The postoperative setting complicates the diagnosis of MH due to overlapping features with other acute conditions. Tachycardia, hypertension, diaphoresis, acidosis, and altered mental status are common to hypertensive crisis, hypoxemia, hypercarbia, sepsis, thyroid storm, NMS, serotonin syndrome, and myocardial infarction (MI), with no single finding pathognomonic for MH. However, the patient’s rapid symptom progression, arterial blood gas showing combined metabolic and respiratory acidosis, and notably the clear clinical improvement following dantrolene administration suggest that MH could not be excluded. Additional limitations for this case include the lack of definitive testing to confirm MH. Moving forward, confirmatory testing with caffeine halogen, genetic testing, or muscle biopsy was recommended for definitive diagnosis and future anesthetic planning. Additionally, the patient remained normothermic and had no muscle rigidity, and CK was normal, which would typically be present in MH.

Finally, other cases from the available literature underscore that delayed or postoperative MH can occur outside the classic intraoperative window. A case in 2021 describes a 77-year-old man who developed MH signs - including masseter rigidity, tachypnea, and hyperthermia - shortly after arrival in the recovery room, with rapid improvement after dantrolene administration [7]. Similarly, a markedly delayed case occurred 11 hours after routine tonsillectomy and adenoidectomy, presenting with tachycardia, hyperthermia, and metabolic acidosis, again resolving with prompt dantrolene treatment [8]. In another postoperative case following breast cancer surgery, MH was confirmed with contracture testing, and rapid recognition with immediate dantrolene was life-saving [9]. Collectively, these cases demonstrate that the latency between anesthetic exposure and clinical presentation may be longer than traditionally expected, and vigilance for MH should extend well into the postoperative period - even in elderly patients with atypical profiles.

## Conclusions

This case underscores the importance of maintaining a broad differential diagnosis when evaluating postoperative patients presenting with acute hypermetabolic deterioration. While sepsis, endocrine emergencies, and neuroleptic syndromes must be considered, malignant hyperthermia should remain on the differential even in elderly patients, those without a suggestive history, and with delayed symptom onset. In addition, post-discharge MH testing should be done in these patients to guide future anesthetic planning. Ultimately, the patient’s rapid improvement following dantrolene administration reinforces the principle that MH cannot be definitively ruled out in such cases. Prompt recognition, empiric treatment, and continued vigilance throughout the postoperative period are essential to minimize morbidity and mortality.
